# Impact of Time and Handwashing on Infection Inference in Scenario‐Based Exposures Among Adults and Children in Japan

**DOI:** 10.1002/puh2.226

**Published:** 2024-08-13

**Authors:** Fumikazu Furumi, Yumiko Nishio

**Affiliations:** ^1^ Department of Education Shizuoka University Shizuoka Japan; ^2^ Department of Education Kio University Nara Japan

**Keywords:** communicable diseases, handwashing, infection inference, preschooler

## Abstract

**Introduction:**

The COVID‐19 pandemic has underscored the importance of preventive measures like handwashing and mask‐wearing. However, preschoolers often struggle to comprehend disease transmission. This study examined how time and handwashing impact adults’ and preschoolers’ inferential processes related to infectious disease transmission.

**Methods:**

Thirty‐eight Japanese adults aged 18–23 years (8 men; 30 women) and 36 Japanese children aged 5–6 years (15 boys; 21 girls) participated in this study. Participants were presented with scenarios involving an infectious individual who was present (1) at the same time, (2) immediately before, or (3) the previous day. Scenarios were presented via PowerPoint for adults and as a picture‐story for preschoolers, with questions on infection likelihood pre‐ and post‐handwashing.

**Results:**

Both age groups perceived a significantly higher likelihood of infection for same‐time exposure than for other exposures. Preschoolers estimated a lower likelihood of infection than adults ($\eta_{p}^{2}$ = 0.25). Handwashing significantly reduced perceived infection risk, particularly for same‐time ($\eta_{p}^{2}$ = 0.34) and immediately after ($\eta_{p}^{2}$ = 0.10) exposures.

**Conclusion:**

This study highlights the critical need for age‐appropriate communication about infection prevention, particularly for young children. Preschoolers exhibited a more positive perception of infectious disease transmission, which necessitates reinforcing multiple preventive measures beyond handwashing. The findings underscore the importance of considering cognitive development and the influence of contemporary experiences like the COVID‐19 pandemic when educating about disease transmission. Future research should further explore the relationship between cognitive development, individual differences, and infection reasoning to enhance public health strategies for varying age groups.

## Graphical abstract

This study explored how time and handwashing influence infection risk perception in Japanese adults and preschoolers. Adults and preschoolers both saw higher infection risk in same‐time scenarios, but preschoolers were more optimistic than adults. Guidance on infection prevention should consider this optimistic bias in children.

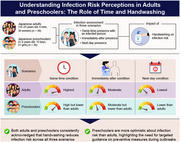

## 1 Introduction

Our social lives expose us to various infectious diseases, including influenza and COVID‐19. The impact of these diseases has grown significantly, particularly with the global spread of the COVID‐19 pandemic. Several preventive measures, such as wearing masks [1] and handwashing [2], have proven effective in curbing the transmission of COVID‐19. Understanding disease infection risks and taking preventive measures based on such estimates can mitigate the social effects of infectious diseases. However, numerous studies have demonstrated that preschoolers have difficulty comprehending disease transmission and avoiding contaminants [3, 4, 5]. Preschoolers have difficulty predicting who might fall ill and how to avoid contamination [6]. Examining the differences between preschoolers and adults in how they reason disease transmission is important, particularly after the COVID‐19 pandemic; thus, effective ways to communicate infection prevention strategies to preschoolers should be established.

Regarding disease transmission, it is indicated that preschoolers aged 4–5 tend to perceive not only colds but also toothaches and scraped knees as contagious diseases [7]. These findings highlight that preschoolers exhibit difficulty in distinguishing between infectious and noninfectious ailments. For instance, children aged 5–8 struggle to distinguish infectious diseases (e.g., colds and chickenpox) from noninfectious conditions (e.g., atopic dermatitis and toothaches) and injuries (e.g., fractures and lacerations) [8]. Thus, although preschoolers have difficulty distinguishing between infectious and noninfectious diseases [9, 10], these studies show that preschoolers mistakenly recognize contagion even in noninfectious diseases. This difficulty in reasoning about infectious diseases in preschoolers can be attributed, in part, to the fact that bacteria and viruses causing illnesses are invisible to the naked eye [4].

A previous study on how children perceive behaviors that may lead to disease transmission found that, by age 7, children can predict the risks associated with illnesses based on information about disease type (infectious vs. noninfectious), level of contact (changes in physical contact), and frequency of contact [11]. Similarly, children aged 3–6 made about disease transmission by focusing on the degree of contact between a protagonist and a character with an illness [12]. The results indicated that children understood that there was a greater risk of infection whenever they were in close proximity to or interacted with someone who was ill. However, these studies did not explore the possibility of viruses lingering in the air even after the infected individuals had left the location.

The existence of asymptomatic carriers has been highlighted during the COVID‐19 pandemic, leading to widespread transmission. The risk of infection through aerosol particles and contact with contaminated surfaces has also been identified [13]. Therefore, understanding how preschoolers comprehend the presence of viruses released by infected individuals and the extent of contamination over time is critical. This study developed scenarios in which an adult/preschooler was present where an individual suspected of infection (1) was present, (2) was present a few minutes ago, and (3) was present the day before. In this study, we proposed three hypotheses to examine how adults and preschoolers make inferences regarding disease transmission by considering changes in contamination levels over time and due to the presence of a virus. As preschoolers tend to exhibit a positive bias when considering the future [14, 15], Hypothesis 1 posited that, unlike adults, preschoolers would not predict infection, even in situations where infection was likely to occur. Additionally, owing to the observed decrease in the infectivity of COVID‐19 within approximately 20 min [16], Hypothesis 2 proposed that both adults and preschoolers would predict a higher likelihood of infection when noninfected individuals are present at the same time and place as an infected person, compared to when they are present at the location shortly before or the day after the infected person's presence. Furthermore, considering the widespread emphasis on frequent handwashing for preventing infection in early childhood education during the COVID‐19 pandemic [17], Hypothesis 3 aimed to compare how adults and preschoolers recognized the effects of handwashing.

## 2 Materials and Methods

### 2.1 Participants, Sampling Method, and Data Collection

The required sample size was determined by conducting a power analysis, using G*power 3 [18], prior to data collection. The analysis indicated that 36 participants per group, totaling 72 participants, would be appropriate (*α* = 0.05, power = 0.80). Thus, the experimental study included 38 Japanese university students and 36 Japanese preschoolers, attending a university‐affiliated kindergarten. Data were collected in Shizuoka city, which has a population of over 650,000 people. All participants were selected through random sampling. The inclusion criteria for the study participants were being Japanese nationals and native speakers of Japanese. The exclusion criterion was having a mental illness. We collected data from September to November 2022. All adult participants provided informed consent and participated in this study in a quiet room at the university. For preschoolers, we obtained informed consent from the kindergarten director rather than the participants’ parents, as the university‐affiliated kindergarten is a research field for the institution.

### 2.2 Materials and Procedures

We presented three scenarios to adult participants using Microsoft PowerPoint on a personal computer (LAPTOP with Windows 10). For preschool participants, the scenarios were presented as picture‐story cards with hand‐drawn images. Questions regarding disease infection and infection after handwashing were posed for each scenario. The three scenarios were presented in a counterbalanced order among participants.

#### 2.2.1 Scenario 1: Same‐Time Exposure

##### 2.2.1.1 Same‐Time Exposure (Adults)

Fumio is at the university. Unfortunately, Fumio has not been feeling well since the morning and has a fever, runny nose, and cough. Zenji comes and sits beside him. They attend the class together.

##### 2.2.1.2 Same‐Time Exposure (Preschoolers)

Fumio has not been feeling well since the morning. He has a fever, runny nose, and cough. Despite this, Fumio is playing in the playroom. Zenji joins Fumio, and they play together in the same place.

#### 2.2.2 Scenario 2: Immediately After Exposure

##### 2.2.2.1 Immediately After Exposure (Adults)

Kazuyo is at the university. Unfortunately, Kazuyo has not been feeling well since the morning. She has a fever, runny nose, and cough. After attending one class, Kazuyo leaves. Immediately after that, Machiko enters the classroom and sits in the seat Kazuyo was sitting in.

##### 2.2.2.2 Immediately After Exposure (Preschoolers)

Youko has not been feeling well since the morning. She has a fever, runny nose, and cough. Despite this, Youko is playing in the playroom. Subsequently, Youko's mother comes to pick her up. Immediately after this, Kazuko plays in the same place where Youko was playing.

#### 2.2.3 Scenario 3: Next‐Day Exposure

##### 2.2.3.1 Next‐Day Exposure (Adults)

Eiko is at the university. Unfortunately, Eiko has not been feeling well since the morning. She has a fever, runny nose, and cough. After attending one class, Eiko leaves. The next day, Manabu enters the classroom and sits where Eiko sat the day before.

##### 2.2.3.2 Next‐Day Exposure (Preschoolers)

Kazuo has not been feeling well since the morning. He has a fever, runny nose, and cough. Despite this, Kazuo is playing in the playroom. Subsequently, his mother comes to pick him up. The next day, Manabu plays in the same place where Kazuo played the day before.

#### 2.2.4 Procedures

Following each scenario, the participants were asked about the likelihood of the protagonist becoming ill. First, they were asked whether the protagonist would become ill (e.g., “Will Zenji become ill or not after this?”); thereafter, they were asked whether their answer was “definitely” or “probably.” Handwashing was introduced after the protagonists in each scenario returned home (e.g., “After returning home, Zenji washed his hands”). Subsequently, the participants were asked whether the protagonist was likely to become ill (e.g., “Will Zenji, who washed his hands, become ill after this or not?”), and afterward, they were asked whether their answer was “definitely” or “probably.”

#### 2.2.5 Scoring and Data Analysis

Responses scored with “definitely will not get ill” received zero points, “probably will not get ill” received one point, “probably will get ill” received two points, and “definitely will get ill” received three points [12]. A three‐way analysis of variance was conducted for the response scores using age group (adults and preschoolers) as the between‐participants factor and time point (same time, immediately after, and next day) and handwashing (before handwashing and after handwashing) as within‐participants factors. There was a significant main effect of age group, *F*(1, 72) = 24.07, *p* < 0.001, $\eta_{p}^{2}$ = 0.25, with adults scoring higher than preschoolers. The main effect of time point was also significant, *F*(2, 144) = 23.80, *p* < 0.001, $\eta_{p}^{2}$ = 0.25. All analyses were performed using IBM SPSS Statistics 25.

## 3 Results

The participants’ basic demographic characteristics are presented in Table 1. Post hoc comparisons demonstrated significant differences between the same‐time and immediately after exposure, *t*(72) = 5.24, *p* < 0.001, *r* = 0.53, as well as the same‐time and next‐day conditions, *t*(72) = 6.00, *p* < 0.001, *r* = 0.58, with higher scores observed in the same‐time condition. Furthermore, there was a significant main effect of handwashing, *F*(1, 72) = 25.80, *p* < 0.001, $\eta_{p}^{2}$ = 0.26, with higher scores observed in the before‐handwashing condition than in the after‐handwashing condition. Additionally, the interaction between time point and handwashing was significant, *F*(2, 144) = 6.08, *p* = 0.004, $\eta_{p}^{2}$ = 0.08. The simple main effects analysis indicated that handwashing has a significant effect in the same‐time, *F*(1, 216) = 37.02, *p* < 0.001, $\eta_{p}^{2}$ = 0.34, and immediately after *F*(1, 216) = 8.07, *p* = 0.005, $\eta_{p}^{2}$ = 0.10, conditions. Table 2 shows the means and standard deviations of scenario task scores.

**TABLE 1:** Demographic data of the participants of a study on disease transmission perceptions (Shizuoka, Japan, September–November 2022).

	Sex	Mean age (years)	Age range (years)
University students	8 men 30 women	20.60 (SD = 1.19)	18–23
Preschoolers	15 boys 21 girls	5.72 (SD = 0.45)	5–6

**TABLE 2:** Means and standard deviations of adults’ and preschoolers’ scenario task scores in a study on disease transmission perceptions (Shizuoka, Japan, September–November 2022).

		Before washing hands	After washing hands
Preschoolers	Same time	1.42 (SD = 0.95)	0.58 (SD = 0.83)
	Immediately after	0.86 (SD = 0.92)	0.50 (SD = 0.73)
	Next day	0.83 (SD = 0.90)	0.53 (SD = 0.87)
Adults	Same time	1.79 (SD = 0.47)	1.34 (SD = 0.47)
	Immediately after	1.32 (SD = 0.52)	1.08 (SD = 0.35)
	Next time	1.08 (SD = 0.48)	0.90 (SD = 0.38)

## 4 Discussion

This study revealed a significant main effect of age group, indicating that preschoolers estimated a lower overall susceptibility to infectious diseases than adults. This supported Hypothesis 1, which posited that preschoolers would not infer infections even in situations where infection was likely to occur. Furthermore, this finding is consistent with prior research, which suggested that preschoolers tend to have a more optimistic and positive attitude when inferring the future likelihood of infectious disease transmission [14, 19, 20]. In addition, our findings revealed that preschoolers exhibit more optimism than adults in estimating the rate of recovery from illness; this is consistent with the findings of a previous study [15], which suggested that children attribute more positive future outcomes to all cures. According to the participants of both age groups, the risk of infection was the lowest in the next‐day and after‐handwashing conditions. Only 5 of 38 adults and 23 of 36 preschoolers responded with “definitely will not get ill” in this condition. Therefore, preschoolers tended to perceive infection risks more optimistically than adults.

Furthermore, the main effect of the time point factor was significant, with the same‐time condition yielding higher scores than the immediately after and next‐day conditions. This finding supported Hypothesis 2, which posited that both adults and preschoolers would infer a higher likelihood of infection when present near a suspected infectious individual at the same time, as opposed to being present in the location where a suspected infectious individual was present immediately before or the day before. During post‐debriefing, adult participants reported that they believed that “after one day, the virus would be largely inactive,” and that the next‐day condition would involve better ventilation and disinfection, resulting in a lower perceived risk of infection.

During the period in which this study was conducted (2022), Japan was still significantly impacted by COVID‐19. As such, many of the study participants presumed COVID‐19 to be the infectious disease referred to in the scenarios, owing to the infectivity of COVID‐19 reducing to approximately 10% after 20 min [16]. Consequently, participants may have associated the diminishing potency of the virus over time with the effects of ventilation and disinfection, which may have resulted in predictions that the risk of infection would be lower in the immediately after and next‐day conditions than in the same‐time condition.

Additionally, the same‐time and immediately after conditions showed higher scores in the before‐handwashing condition than in the after‐handwashing condition. This finding partly supported Hypothesis 3, which posited that both adults and preschoolers would recognize the effectiveness of handwashing, thereby leading to a lower likelihood of infection after handwashing across all conditions. The participants’ reasoning was influenced by their knowledge of fundamental infection control measures, such as mask‐wearing [1] and handwashing [2]. The disparity between the scores in the before‐ and after‐handwashing conditions was significant only in the same‐time and immediately after conditions, not in the next‐day condition. This may be due to both adults and preschoolers inferring that an individual “definitely will not get ill” in the next‐day condition, even before handwashing. In the next‐day condition, 32 of 38 adults and 30 of 36 preschoolers responded with either “probably will not get ill” or “definitely will not get ill” in the before‐handwashing condition. Thus, there was no significant difference between the before‐ and after‐handwashing scores.

This study investigated variations in the risk of infection based on the degree of contact with individuals suspected of having infections by employing a scoring method based on a previous study [12]. In contrast to the findings of the previous study [12], this study exhibited relatively lower scores among preschoolers. This divergence could be attributed to the contrast in story descriptions. The previous study [12] explicitly mentioned “touch each other,” but this study chose “playing next to each other” to align with the narratives for adults.

Moreover, this study elucidated the disparities in infection reasoning between adults and preschoolers, revealing how both age groups alter their inferences regarding infection transmission based on whether healthy individuals were in the presence of individuals suspected of being infected at the same time or were present in the location individuals suspected of being infected were present immediately before or the day before. The results further revealed that both adults and preschoolers modify their inferences regarding the risk of infection in the before‐ and after‐handwashing conditions. The infectious disease referred to in the scenarios, such as COVID‐19 or influenza, was not specified; however, considering that the study was conducted in 2022, many participants may have had COVID‐19 in mind when responding to the questions. This is worth noting because certain infectious diseases may exhibit variations in the risk of infection and the rate of progression; for instance, the infectivity of COVID‐19 diminishes to approximately 10% within 20 min [16], whereas the influenza virus survives for 6–8 h under specific conditions [21]. Additionally, asymptomatic individuals may contribute to the spread of COVID‐19 [22], indicating that different infectious diseases have varying risks of infection and symptomatology. Moreover, the extent of knowledge possessed by participants regarding COVID‐19's declining infectivity over time [16] remains uncertain, and this study could not explore individual differences in the knowledge of infectious diseases.

### 4.1 Limitations

This study revealed discrepancies in the inferences made regarding infectious disease transmission between adults and preschoolers; however, a limitation of this study is that it could not ascertain the cognitive development responsible for these differences or the relationship between response tendencies and individual variations. Inferences have been associated with working memory [23, 24], which develops in early childhood [25]. Thus, preschoolers’ reasoning about infections could be intertwined with their working memory. However, infection‐related inferences may also be associated with preschoolers’ optimism and positive thinking [14]. Therefore, future research should focus on elucidating the connection between individual differences, cognitive development, and infection reasoning when it comes to infectious diseases.

## 5 Conclusion

This study investigated the conjectures of adults and preschoolers regarding infectious disease transmission experimentally, considering the effects of time and handwashing efficacy regarding infectious diseases. Preschoolers estimated a lower overall susceptibility to infection. This observation aligns with the insight that preschoolers tend to be optimistic when considering the future, emphasizing the significance of infection prevention among preschoolers during periods of disease outbreaks. Preschoolers tend to believe that handwashing alone renders them “unlikely to contract the illness.” In situations where relying solely on handwashing is inadequate, highlighting other infection control measures, along with communicating that handwashing is not an absolute safeguard, is crucial.

## Author Contributions

**Fumikazu Furumi:** writing–original draft (lead), writing–review and editing (lead), conceptualization (lead), methodology (lead), data curation (lead), formal analysis (lead), funding acquisition (lead), investigation (lead), resources (lead), software (lead), validation (lead), project administration (lead). **Yumiko Nishio:** writing–review and editing (supporting), conceptualization (supporting), methodology (supporting), visualization (lead).

## Ethics Statement

This study was conducted with approval from the Ethics Committee of Research Involving Human Subjects at Shizuoka University (approval number: 22‐27).

## Consent

All adult participants provided informed consent. For preschoolers, we obtained informed consent from the kindergarten director rather than from the participants’ parents, as the university‐affiliated kindergarten is a research field for the institution. Additionally, the Ethics Committee of Research Involving Human Subjects at Shizuoka University specified that informed consent can only be obtained from the kindergarten director. Preschoolers participated in this study in a quiet room at the kindergarten.

## Conflicts of Interest

The authors declare no conflicts of interest.

## Data Availability

The data that support the findings of this study are openly available in Open Science Framework at: https://osf.io/af9h8/. The materials necessary to replicate the findings are not publicly accessible but may be shared by the corresponding author. The analyses presented here were not preregistered.
